# A Comprehensive Evaluation of the HPV Neutralizing Antibodies in Guangzhou, China: A Comparative Study on Various HPV Vaccines

**DOI:** 10.3390/vaccines12111286

**Published:** 2024-11-17

**Authors:** Renyun Zha, Conghui Liao, Daner Lin, Lixuan Zhao, Yanfang Chen, Lin Yao, Xiaokang Li, Boyang Yi, Ting Li, Jianpeng Xiao, Yan Hu, Zeliang Chen, Cheng Guo, Jianyun Lu, Jiahai Lu

**Affiliations:** 1 School of Public Health, Sun Yat-Sen University, Guangzhou 510080, Chinachenzliang5@mail.sysu.edu.cn (Z.C.); guoch36@mail.sysu.edu.cn (C.G.); 2 One Health Center of Excellence for Research & Training, Sun Yat-Sen University, Guangzhou 510080, China; 3 Guangzhou Baiyun District Center for Disease Control and Prevention, Guangzhou 510445, China; 4 Translational Cancer Research Center, Peking University First Hospital, Beijing 100034, China; 5 School of Laboratory Medicine and Life Science, Wenzhou Medical University, Wenzhou 325000, China; 202111115611003@zcmu.edu.cn; 6 Guangdong Provincial Institute of Public Health, Guangdong Provincial Center for Disease Control and Prevention, Guangzhou 511430, China; 7 Department of Obstetrics and Gynecology, The First Affiliated Hospital of Wenzhou Medical University, Wenzhou 325000, China; 8 National Medical Products Administration Key Laboratory for Quality Monitoring and Evaluation of Vaccines and Biological Products, Guangzhou 510080, China; 9 Hainan Key Novel Thinktank “Hainan Medical University ‘One Health’ Research Center”, Haikou 571199, China; 10 Research Institute of Sun Yat-Sen University in Shenzhen, Shenzhen 518057, China; 11 Key Laboratory of Tropical Diseases Control, Sun Yat-Sen University, Ministry of Education, Guangzhou 510080, China; 12 Institute of One Health, Wenzhou Medical University, Wenzhou 325000, China

**Keywords:** HPV vaccine, neutralizing antibody, seroprevalence, pseudovirion

## Abstract

Background: The evaluation of HPV vaccine effectiveness is essential for informing public health strategies, yet there remains a gap in understanding humoral immune responses generated by different HPV vaccine formulations in regional populations. This study addresses this gap by evaluating the immunogenicity of the newly developed HPV vaccine Cecolin (Wantai), alongside various imported vaccines, including bivalent, quadrivalent, and nonavalent options available in China. Methods: From March 2023 to June 2024, a total of 352 participants were enrolled, including 87 females aged 9–14 years who received two doses of the bivalent HPV vaccine (Cecolin), 215 females aged 15–45 years who were fully vaccinated with various HPV vaccines, and 50 non-recipients. Follow-up assessments were conducted at six timepoints during the administration of Cecolin. Serum was collected at enrollment and at each follow-up visit for antibody assessments using a pseudovirion-based neutralization assay (PBNA). Findings: The longitudinal follow-up of females aged 9–14 years revealed a 100% conversion rate for neutralizing antibodies against HPV types 16 and 18 after the second dose, compared to 94.3% and 97.1% conversion rates six months after the first dose. Compared to participants who received full doses of quadrivalent and nonavalent vaccines, females who received two or three doses of Cecolin exhibited higher neutralizing antibody geometric mean titers (GMTs) and non-vaccine-type (HPV31 and HPV33) antibody seroconversion rates. Interpretation: The domestically produced HPV vaccine Cecolin in China demonstrates strong immunogenicity and holds promise for the large-scale vaccination of females in developing countries to prevent cervical cancer.

## 1. Introduction

Cervical cancer, the fourth most common cancer in women globally with about 660,000 new cases and 350,000 deaths in 2022, has the highest incidence and mortality rates in low- and middle-income countries [1]. It has been proven that among all carcinogenic factors, persistent infection with human papillomavirus (HPV) contributes most significantly to the development of cervical cancer [2]. HPV types 16 and 18 are together responsible for 71% of cervical cancer cases and a substantial fraction of other cancers attributable to HPV infections, including those of the anogenital tract (vulvar, vaginal, anal, and penile), as well as the head and neck (oropharynx, oral cavity, and larynx) [3]. Twelve high-risk HPV genotypes, including HPV16, 18, 31, 33, 35, 39, 45, 51, 52, 56, 58, and 59, pose carcinogenic risks [4]. The distribution of HPV types across different regions worldwide exhibits minor variations. For instance, aside from HPV type 52 being most prevalent in Taiwan, Japan, and East Africa, type 16 predominates in other regions with the highest prevalence rates [5]. While HPV16, 31, and 18 are frequently reported as predominant in European nations, two studies carried out in the northern and southern parts of China show that HPV16, 52, and 58 are the most common high-risk infection types among Chinese women [6,7]. Due to the persistent infection of HPV, which is a primary cause of invasive cervical squamous cell carcinoma (ICC), the prevention of cervical cancer primarily relies on HPV vaccination, along with the detection and treatment of precancerous lesions before the progression to cancer [8,9]. In November 2020, the World Health Organization (WHO) launched the “Global Strategy to Accelerate the Elimination of Cervical Cancer as a Public Health Problem”, recommending a comprehensive approach to prevent and control cervical cancer, and HPV vaccination is advocated as the most direct, effective, and cost-effective preventive measure [10].

Since the introduction of the first HPV vaccine in 2006, there are currently bivalent, quadrivalent, and nonavalent (depending on the HPV antigens included) vaccines available worldwide [11]. China has licensed two domestic bivalent HPV16 and 18 vaccines (Cecolin^®^ [Xiamen Innovax, Xiamen, China] and Walrinvax^®^ [Yuxi Zerun Biotech, Yuxi, China]) and three imported vaccines, including a bivalent HPV16 and 18 vaccine (Cervarix^®^ [GlaxoSmithKline Biologicals, Wavre, Belgium]), a quadrivalent HPV6, 11, 16, and 18 vaccine (Gardasil^®^ [Merck Sharp & Dohme, Kenilworth, NJ, USA]), and the world’s only second-generation HPV vaccine against HPV types 6, 11, 16, 18, 31, 33, 45, 52, and 58 (Gardasil 9^®^ [Merck Sharp & Dohme, USA]), providing options for HPV vaccination for the Chinese population.

The Escherichia coli-produced bivalent HPV vaccine (Cecolin 2), characterized by low-cost and high-yield production, has been proven to be well tolerated and highly efficacious against HPV16- and 18-associated high-grade genital lesions and persistent infection in a phase 3, double-blind, randomized, controlled trial [12]. Long-term studies conducted in China for the bivalent vaccine (Cervarix 2) and the quadrivalent vaccine (Gardasil 4) demonstrated vaccine efficacy against HPV16/18-associated CIN [13,14]. The nonavalent HPV vaccine (Gardasil 9) provided the most protective antibodies against the nine vaccine-targeted HPV types for approximately 10 years after three doses of vaccination in boys and girls aged 9 to 15 years, as well as sustained immunogenicity and effectiveness [15].

In August 2020, the first free government-led HPV vaccination campaign in China was launched in Ordos City, Inner Mongolia. Guangdong Province then became the first province nationwide to implement free HPV vaccination for eligible school-aged girls across the entire province, with the Guangzhou municipal government funding the free administration of the bivalent HPV vaccine Cecolin 2 to middle school girls aged 9–14. However, the lack of early domestic trial data in China led to a delay in the market entry of HPV vaccines in the country [16]. Consequently, given the extensive use of various HPV vaccinations and their popularization among younger age groups in China, assessments of the effectiveness and side effects of multi-brand vaccines customized for the local population are required. Research on the immunogenicity of the domestic bivalent HPV vaccine, especially concerning HPV neutralizing antibodies which are crucial for preventing cervical cancer, is limited [17]. Although clinical trials have shown that Cecolin has non-inferior immunogenicity and safety to that of Gardasil, it is limited to ELISA and a subset of participants for PBNA analysis, lacking full immune process analysis of non-vaccine-type- and vaccine-type-specific neutralizing antibodies [18]. Currently, various HPV vaccines have been employed in China, and further research is required to investigate the efficacy and cross-protection effects of different vaccine types, particularly domestically produced bivalent vaccines [19]. In this study, we enrolled fully immunized females in Guangzhou City, who received three valent types (bivalent, quadrivalent, and nonavalent) of HPV vaccines produced by three different manufacturers (Xiamen Wantai, GlaxoSmithKline, and Merck). For the purpose of evaluating the HPV vaccines that are applicable in China, we employed the pseudovirion-based neutralization assay (PBNA), which is the gold standard recommended by the WHO, to identify neutralizing antibodies (NAbs).

## 2. Materials and Methods

### 2.1. Study Participants

This study enrolled middle school girls aged 9–14 years from Baiyun District, Guangzhou, Guangdong Province, who were administered two doses of the bivalent HPV vaccine (Cecolin 2). Follow-up was conducted at 6 timepoints across 2 stages before and after vaccinations (Stage 1, Day 0, Day 14, and Day 28, and Stage 2, Day 0, Day 14, and Day 28, with a 6-month interval between Stage 1 and Stage 2). Female volunteers aged 15–45 years who were fully vaccinated with HPV vaccines were recruited as well. Serum was collected at enrollment and at every follow-up visit for antibody assessments by PBNA. Inclusion criteria include the following: (1) vaccination records are accessible in the Guangdong Provincial Vaccination System, and (2) the immunization schedule has been completed. Exclusion criteria include pregnancy and previous or ongoing immunotherapy.

### 2.2. Pseudovirion-Based Neutralization Assay (PBNA)

The production of the nine-type HPV pseudovirion (HPV6, HPV11, HPV16, HPV18, HPV31, HPV33, HPV45, HPV52, HPV58) was achieved through the co-transfection of modified HPV L1L2 expressing plasmids and reporter plasmids into HEK293FT cells (RRID: CVCL_6911), which has been described previously, with modification [20]. HPVL1L2 expressing plasmids include p16sheLL (RRID: Addgene_37320) [21], p18sheLL (RRID: Addgene_37321) [22], p6sheLLr (RRID: Addgene_37318) [23], p31sheLL (RRID: Addgene_37322) [22], p45sheLL (RRID: Addgene_37323) [22], p52sheLL (RRID: Addgene_46950) [24], p58sheLL (RRID: Addgene_37324) [25], pVITRO-HPV11L1L2 (RRID: Addgene_52590) [26], and pVITRO-HPV33 L1L2 (RRID:Addgene_52493) [26]. Reporter plasmids include the enhanced green fluorescent protein reporter plasmid pCMV-C-EGFP [27] and the red fluorescent protein (RFP) reporter plasmid pRwB (RRID: Addgene_48734) [28]. Briefly, 293FT cells that were maintained in growth medium (high-glucose Dulbecco’s modified Eagle’s medium + 10% fetal bovine serum + 1% Penicillin–Streptomycin solution) were co-transfected with a HPV L1L2 expressing plasmid together with a reporter plasmid, using Lipofectamine 2000 (Invitrogen), according to the manufacturer’s instructions. After 48 h of incubation at 37 °C with 5% CO2, the cells were trypsinized and lysed with cell lysis buffer (0.5% Triton X-100, 0.1% Benzonase, and 0.1% Plasmid Safe in Dulbecco’s phosphate-buffered saline solution) for 24 h. High-fluorescence intensity and cell density were detected under a fluorescent inverted microscope. The lysates were then centrifuged at 4 °C overnight using PEG-it solution (System Biosciences) for purification. Pseudovirion combinations of both red and green reporters were diluted to 200TCID50 (50% tissue culture infective dose) and mixed with serum dilutions ranging from 1:40 to 1:125,000, followed by incubation at 4 °C for 1 h before being transferred into pre-seeded 96-well cell culture plates containing 293FT cells. Virus and negative control wells (DMEM medium) were included. After 72 h of incubation, fluorescent spots were counted using the Fluoro-Immuno-SPOT Analyzer (ImmunoSpot S6 Ultra, Cellular Technology Ltd., Shaker Heights, Ohio (OH), USA), and the serum neutralization titers were defined as the 50% maximal inhibitory concentration, calculated with the Reed–Muench method [29].

### 2.3. Statistical Analysis

Groups were compared using Bonferroni’s multiple comparisons test as appropriate. *p* values less than 0.05 were considered significant. All statistical tests were two-sided. HPV-type-specific seroprevalence (percentage of neutralization-positive serums among all serums) and geometric mean titers (GMTs, neutralization-positive serums only) with 95% confidence intervals (95% CIs) were calculated using GraphPad Prism 9. All statistical analyses with significant differences in figures in this study are presented in Supplementary Table S1.

## 3. Results

### 3.1. Study Population

**Figure 1:**
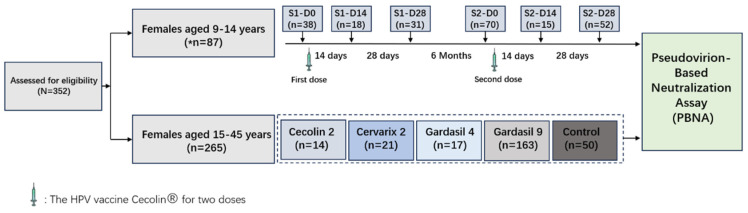
Study design. This is a study that combines longitudinal and cross-sectional analysis, including samples of females aged 9–14 years and females aged 15–45 years who received four types of HPV vaccines. A total of 352 participants and 489 corresponding serum samples were included in this study. * Not all the girls were included before the first dose; some were included before the second dose.

From March 2023 to June 2024, a total of 352 participants were enrolled, including 87 females aged 9–14 years and 265 females aged 15–45 years. A total of 489 serum samples, including 50 serum samples from the control group (without receiving any HPV vaccine), 238 samples from the recipients of Cecolin 2 (87 girls with 224 samples and 14 adults with 14 samples), 21 samples from the recipients of Cervarix 2, 17 samples from the recipients of Gardasil 4, and 163 samples from the recipients of Gardasil 9, were collected. The participants’ vaccination information, including age, the number of doses administered, and the timepoints of all doses, was under consideration (Table 1). Compared to the six follow-up visits for girls, only one serum collection was conducted for adults after HPV vaccinations (Figure 1). The HPV vaccine Cecolin 2 for girls had a schedule of two doses with a 6-month interval, with follow-up starting before the immunization. Nineteen girls completed the main sample collection procedures for NAbs analysis in this study.

### 3.2. Humoral Immune Response

**Figure 2:**
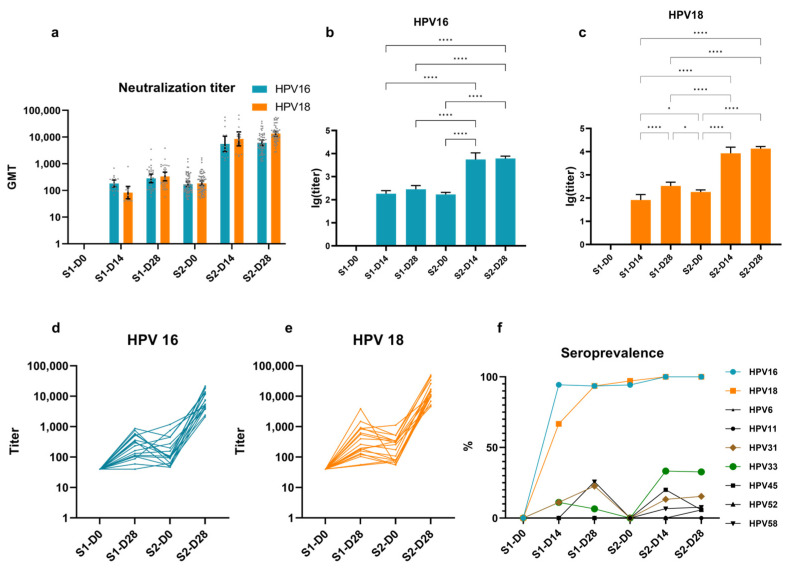
Comparisons of HPV-type NAbs across different stages of the cohort. (**a**) The HPV16 and 18 NAb GMTs with 95% CI of the girls in different stages. (**b**) The HPV16 and (**c**) HPV18 NAb mean lg (titer) with 95% CI and significant difference comparisons. (**d**) The trend in HPV16 and (**e**) HPV18 NAb titers among the 19 girls who completed the primary follow-up timepoints. (**f**) The seroprevalence of HPV-type NAbs of the girls vaccinated with Cecolin 2 throughout the whole follow-up. To better illustrate the pattern of trends, the negative points (titer < 40) of S1 are represented by 40 in (**d**,**e**). The negative samples are not included in the PBNA-GMT. PBNA NAbs’ positive reference value: titer ≥ 40. The gray dots represent individual participant titers; bars depict geometric mean titers with exact 95% confidence intervals. GMT: geometric mean titer. Lg (titer): log-transformed titer. *: *p* < 0.05; ****: *p* < 0.0001. Considering the use of the multiple comparisons test, the *p* values have been adjusted (the family-wise alpha threshold and confidence level were 0.05 and 95%, same below).

As expected, in samples collected from girls in the cohort before receiving their first dose (S1-D0), NAbs were not detected at the minimum threshold (Figure 2a, Supplementary Table S2). The continuous follow-up of this population revealed a modest initial rise in GMTs during S1 (post first dose), followed by a slight decline approaching the beginning of S2 (pre-second dose). Subsequently, a rapid and substantial increase was observed during S2. The comparison of NAb mean lg (titer) at different stages shows significant differences between S1 and S2, along with three timepoints of S2, for both HPV16 and HPV18 (*p* < 0.0001, Figure 2b,c, multiple comparisons test). Additionally, the NAbs of HPV18, rather than those of HPV16, show a significant increase in S1 and a significant decrease between S1-D28 and S2-D0 (*p* = 0.028 and *p* = 0.020, multiple comparisons test). Nineteen girls who completed the primary follow-up period exhibited more pronounced immune trends in HPV16 and HPV18 titers (Figure 2d,e). Nearly all participants showed an “N”-shaped pattern in antibody titers, reaching a nadir at S2-D0 (6 months post first dose) and achieving peak titers at S2-D28. Specifically, compared to the baseline where the NAbs were negative prior to immunization, the HPV16 and HPV18 NAb titers significantly increased 28 days after the first dose (S1-28). However, by six months, the titers decreased to near the critical threshold (titer = 40). The second dose elicited a rapid and high-level response of NAbs, with antibody titers against both HPV types exceeding 1000 in all individuals.

Apart from HPV16 and HPV18, which achieved stable seroconversion rates exceeding 90% by S1-D28, the other seven HPV subtypes exhibited fluctuating seroconversion rates, initially rising and stabilizing at lower levels finally. The serum seroprevalence of non-vaccine-type NAbs against HPV31 (15.4%) and HPV33 (32.7%) was detected as well (Figure 2f, Supplementary Table S2).

No severe adverse events were observed during the follow-up of females aged 9–14 years. Four types of injection site reactions and four types of adverse events were recorded. The most common symptoms included skin swelling, skin itching, and subsequent pain (Supplementary Table S3).

A Cecolin 2+ subgroup was added to represent the overall immunogenicity of the domestically produced HPV vaccine in Chinese populations. The Cecolin 2+ group consisted of females aged 15–45 years (14 serum samples) who received three doses of Cecolin 2 and girls aged 9–14 years (52 serum samples) who received two doses of Cecolin 2. All vaccine recipients aged 14–45 years were divided into four groups based on the time after the last dose (A: 0.1–12, B:12–24, C: 24–36, D: 36–75 months).

**Figure 3:**
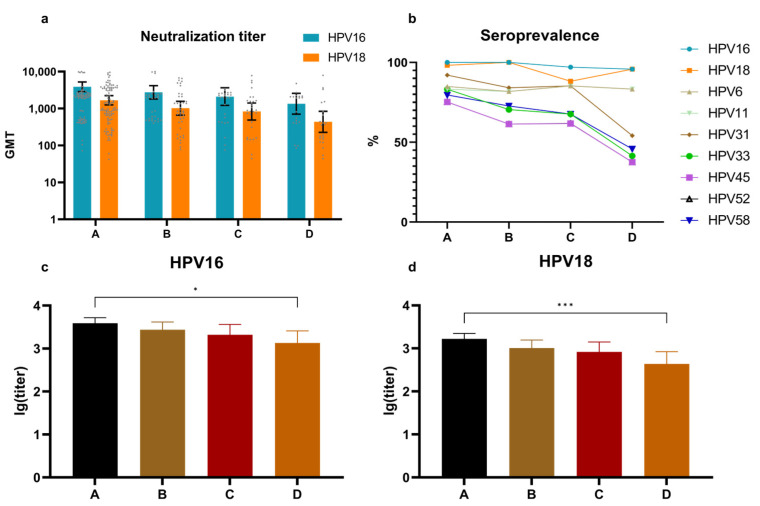
Comparisons of HPV-type NAbs of different periods after vaccination. (**a**) The HPV16 and HPV18 NAb GMTs with 95% CI of the four groups which were classified according to the time of the last dose. (**b**) The seroprevalence of nine HPV-type NAbs of the four groups. (**c**) The HPV16 and (**d**) HPV18 NAb mean lg (titer) with 95% CI and significant difference comparisons of the four groups. The gray dots represent individual participant titers; bars depict geometric mean titers with exact 95% confidence intervals. *: *p* < 0.05; ***: *p* < 0.001.

Based on the NAb GMTs by the timing of the last dose, the antibody levels for HPV16 were consistently higher than those for HPV18 across all time periods, with both showing a decreasing trend over time (Figure 3a). From the perspective of the temporal variation in the NAb seroprevalence of HPV types, the NAbs of HPV types 16, 18, 6, and 11 consistently maintained high levels without showing a decline. However, with the exception of HPV type 31, the NAb seroprevalence of which demonstrated a substantial decrease in Group D, the NAb seroprevalence of the remaining four HPV types exhibited a notable declining trend (Figure 3b). All participants in the control group tested negative (titer < 40); hence, no data are shown. To determine whether NAb titers in women who received the HPV vaccines decreased over time, comparisons of lg (titer) were conducted among all vaccinated groups (Figure 3c,d). The results showed that only group A and group D had significant differences (HPV16: *p* = 0.016, HPV18: *p* = 0.0006, multiple comparisons test).

**Figure 4:**
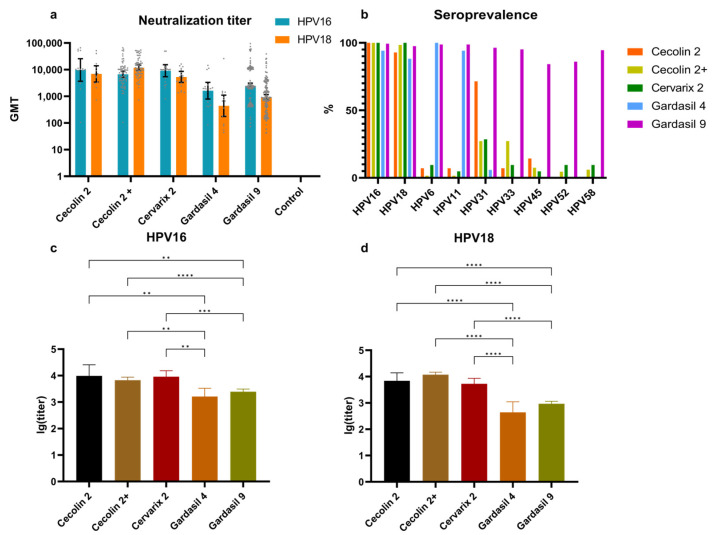
Comparisons of HPV-type NAbs of different HPV vaccine recipients. (**a**) The HPV16 and HPV18 NAb GMTs with 95% CI of the different vaccine type groups and the control group. (**b**) The seroprevalence of nine HPV-type NAbs. (**c**) The HPV16 and (**d**) HPV18 NAb mean lg (titer) with 95% CI and significant difference comparisons. The gray dots represent individual participant titers; bars depict geometric mean titers with exact 95% confidence intervals. **: *p* < 0.01, ***: *p* < 0.001, ****: *p* < 0.0001.

The HPV16 NAb levels were higher than those for HPV18 in all groups except the Cecolin 2+ group that comprised females aged 9–14 and 15–45 (Figure 4a, Supplementary Table S4). In terms of vaccine type, the recipients of the bivalent and nonavalent vaccines had higher levels of NAbs for HPV types 16 and 18 compared to those receiving the quadrivalent vaccine. In addition, the GMTs of the two bivalent vaccine groups were significantly higher than those of the others. The unvaccinated females were all negative for NAbs (titer < 40).

The NAbs against the nine HPV types included in the vaccine were assessed among the recipients of four different vaccines, using a threshold of 40 to determine serum positivity (Figure 4b, Supplementary Table S5). The HPV16 and 18 NAbs of vaccine recipients in the Cecolin 2, Cecolin 2+, Cervarix 2, and Gardasil 9 groups showed nearly 100% seropositivity rates, whereas the Gardasil 4 group exhibited a slight decrease. The NAb seroprevalence for HPV6, 11, 16, and 18 in the Gardasil 4 group was above 88%, while the Gardasil 9 group maintained high seroconversion rates for all types. The two bivalent vaccine groups lacking the HPV31 antigen exhibited seropositivity rates of 22.27% (Cecolin 2+) and 28.57% (Cervarix 2) for the HPV genotype NAbs, respectively. For the HPV33 NAbs, the seropositivity rates differed between the Cecolin 2+ (22.27%) and Cervarix 2 (9.52%) groups. Other types (HPV6, 11, 45, 52, 58) also showed NAb seropositivity rates correspondingly but all below 10%. Furthermore, there were significant differences in the NAb mean lg (titer) among the bivalent vaccine, quadrivalent vaccine, and nonavalent vaccine groups (rather than between the two bivalent vaccine groups), both for HPV16 and HPV18 (Figure 4c,d).

## 4. Discussion

Vaccination against HPV represents the most effective and cost-effective strategy for preventing cervical cancer, significantly reducing global cases and deaths attributed to HPV-related cervical cancer, as well as preventing the incidence of CIN2/3 lesions and their associated costs [30,31]. Bivalent, quadrivalent, and nonavalent HPV vaccines employ insect cell lines or yeast to express HPV capsid protein L1, which self-assembles into virus-like particles (VLPs), devoid of viral genomic material and thereby non-infectious, mimicking the immunogenicity of the native virus [32]. The nonavalent vaccine (Gardasil 9) approved in 2014 contains the most types of VLPs, which can trigger HPV6, 11, 16, 18, 31, 33, 45, 52, and 52 types of antibodies against the virus [33]. However, in China, the per capita cost of administering this vaccine reaches as high as USD 574.71. In contrast, imported quadrivalent (USD 357.21), imported bivalent (USD 262.38), and domestic bivalent HPV vaccines (USD 153.20) may offer a better cost–effectiveness ratio [34]. A study using a validated hybrid model indicates that China’s implementation of the domestically manufactured bivalent HPV vaccine since 2021, transitioning to low-cost nonavalent vaccines by 2031, represents an optimal pathway towards the goal of cervical cancer elimination (fewer than four new cases per 100,000 women) [35]. It is worth noting that, although the price of the nonavalent vaccine is higher than that of other formulations, it targets a broader range of HPV genotypes, providing a wider scope of NAb protection. In contrast, both Cecolin 2 and Cervarix 2 offer a price advantage, but given that they include only two major HPV genotypes, there is a possibility that they may lack protection against other high-risk HPV types. The application and widespread adoption of HPV vaccines also necessitate the consideration of regional viral subtype prevalence, alongside a comprehensive assessment of vaccine efficacy and societal benefits.

Currently, there are three primary serological detection assays applied in HPV vaccine evaluation: virus-like particle–IgG binding assays (VLP-ELISA), competitive (epitope-specific) immunoassays, and the pseudovirion-based neutralization assay (PBNA) [36]. The PBNA is cell-based, labor-intensive, and operationally complex, with low throughput and high cost. However, it theoretically ensures the detection of effective antibodies, thereby guaranteeing methodological neutrality and scientific rigor. The VLP-ELISA, while offering simple operation, high throughput, and low cost, may overestimate vaccine efficacy by detecting the total IgG [37]. This study recruited 87 girls aged 9–14 years who received the HPV bivalent vaccine for whole-process follow-up and 265 women (215 vaccine recipients and 50 unvaccinated) from Guangzhou, China, collecting serum samples and vaccine administration details. The vaccines administered included a domestic vaccine (Cecolin 2) and three previously marketed vaccines (Cervarix 2, Gardasil 4, and Gardasil 9). By detecting neutralizing antibodies through the dual-color fluorescence pseudovirion neutralization assay, we comprehensively analyzed the humoral immune responses induced by each vaccine type.

Previous studies have confirmed that the two-dose vaccination of Cecolin 2 for girls aged 9–14 is non-inferior in immunogenicity to the three-dose vaccination for young women aged 18–26, and our study further contributes evidence to this conclusion [38]. Through monitoring the short-term neutralizing antibody titers during the two-dose administration of the bivalent vaccine, we aim to explore immune patterns and underscore the necessity of completing the vaccination schedule. Furthermore, seroconversion events for non-bivalent vaccine-type antibodies have also been identified. Despite the observed NAb seroconversion for HPV types 31 and 33, these occurrences were unstable and at relatively low levels. Further long-term studies are needed to determine whether genuine cross-protection can be achieved. Due to the cross-sectional nature of antibody assessments following adult group vaccination, we did not collect records of adverse reactions with potential recall bias. However, comprehensive information was obtained from a cohort follow-up of girls, enabling the evaluation of vaccine-associated symptoms. No severe events were observed among all recipients, with adverse reactions limited to common injection site skin swelling and itching.

We found that although all vaccines elicited the desired HPV-subtype NAbs in humans, there were discernible differences in antibody levels. Firstly, recipients in the bivalent vaccine groups exhibited a significantly higher increase in HPV16 and HPV18 antibodies compared to those in the quadrivalent and nonavalent vaccine groups. However, this difference may be attributed to variations in the timing of measurements relative to the final dose administered (rank of time after the last dose: Cecolin 2 < Gardasil 9 < Cervarix 2 < Gardasil 4). Due to the lack of participants administered with bivalent and quadrivalent HPV vaccines, it is difficult to consider the time after the last dose for each group. More subtype neutralizing antibodies and high seroprevalence were observed in the quadrivalent and nonavalent vaccine groups, consistent with previous research findings. Based on a literature review of non-vaccine-type antibodies, it has been shown that the bivalent and quadrivalent vaccines can induce cross-protective antibodies against HPV31, with the bivalent vaccine demonstrating higher efficacy compared to the quadrivalent vaccine [39,40]. However, these cross-protective antibodies against non-vaccine types are only partial and at relatively low levels; thus, they may not persist long term or confer significant protection [41]. Furthermore, we compared the temporal changes in the NAb seroprevalence of nine HPV types and observed that, with the exception of vaccine-type antibodies which remained high, the seroprevalence of other-type NAbs, including HPV type 31, significantly declined. An interesting phenomenon was observed: the GMT of NAbs against HPV16 was consistently higher than that against HPV18 in cross-sectional analyses of vaccine recipients, while in longitudinal comparisons (14 days after the second dose), the situation was reversed. We speculate that this may be due to the relatively short duration of the longitudinal follow-up, which allows for a stronger immune response to HPV18 antibodies initially. Nevertheless, this advantage might be surpassed in the long-term observation of the post-vaccination immune response. This study contributes additional data in exploring the humoral immune responses to various types of HPV vaccines, thereby providing a comprehensive assessment of vaccine efficacy. We observed instances where the recipients of the nonavalent vaccine showed negativity for antibodies against at least two subtypes, with some individuals displaying negativity against up to six subtypes. However, no correlation was observed between antibody negativity and the duration since vaccination (ranging from 10 days to 10.8 years in this study), indicating potential variations in the vaccine immune response attributable to individual factors.

Our study has certain limitations that warrant consideration. Firstly, although immune populations vaccinated with HPV vaccines from three manufacturers across three valency types were recruited for antibody detection, there was a reluctance among the recipients of bivalent and quadrivalent vaccines. Consequently, we obtained a lower-than-expected sample size, diminishing our ability to compare the immunogenicity of different vaccines. Secondly, we enhanced the PBNA method by employing dual-color fluorescence for double-throughput accurate neutralization assays. Nevertheless, this did not alter the cell-based complexity of the testing procedure. Future efforts necessitate simplified, high-throughput, and standardized HPV antibody detection methods for the personalized antibody assessment of vaccine recipients.

## 5. Conclusions

Overall, our study conducted a comprehensive analysis of NAbs in the serum of HPV vaccine recipients in both horizontal and vertical directions, exploring the efficacy and safety in the real world. Both the highly acclaimed nonavalent vaccine and the domestically produced bivalent vaccine which are widely promoted for girls aged 9–14 years in China demonstrate ideal antibody conversion rates for their respective vaccine type. In addition, the higher GMTs and extra cross-protective antibodies of bivalent vaccines compared to high-valent vaccines provide a more cost-effective option for unvaccinated females in developing countries.

## Figures and Tables

**Table 1:** Demographic characteristics and the vaccination of the participants.

	Longitudinal	Cross-Sectional				
	Cecolin 2	Cecolin 2	Cervarix 2	Gardasil 4	Gardasil 9	Control
N	87	14	21	17	163	50
Dose	2	3	3	3	3	-
Age at time of first vaccine dose (years, median) [range] ^①^	12 [12,13]	23 [19,34]	23 [16,39]	24 [15,31]	23 [17,26]	26 ^③^ [24,27]
Time after the last dose (months, mean) [95%CI]	1 ^②^	9.9 [5.9,13.8]	21.2 [12.9,29.5]	37.9 [19.5,56.2]	14.7 [6.7,22.5]	-

95% CI, exact 95% confidence interval; N, number of subjects. ^①^ Participants were aged 9–14 years and 15–45 years at the time of receiving the first dose, and all groups were over 18 years old at the time of sampling, except for the girls receiving Ceoclin 2. ^②^ Samples of S2–D28 from females aged 9–14 years 28 days (≈1 month) after the second dose of Cecolin 2 were included. ^③^ Age at the time of enrollment was recorded.

## Data Availability

The data upon which conclusions are drawn are included in this manuscript or in the supplemental information file provided.

## Supplementary Materials

The following supporting information can be downloaded at https://www.mdpi.com/article/10.3390/vaccines12111286/s1. Table S1: All statistical analyses with significant differences in Figs; Table S2: The GMTs and HPV NAb seroprevalence of the different stages of the cohort; Table S3: Adverse events reported during Days 0-28 post-dose 1, 2 across all vaccination visits; Table S4: The GMTs of HPV16&HPV18 NAbs in the several groups; Table S5: Seroprevalence of nine HPV types NAbs in five vaccine groups; Table S6: Plasmids and Cell used in this study.
