# Supplementary material for: A Comprehensive Evaluation of the HPV Neutralizing Antibodies in Guangzhou, China: A Comparative Study on Various HPV Vaccines
Source: Vaccines (Basel). 2024 Nov 17;12(11):1286. doi: 10.3390/vaccines12111286 (PMC11599073; doi:10.3390/vaccines12111286)
Supplement: Supplementary file 1 [file vaccines-12-01286-s001.zip › vaccines-3285015-supplementary.pdf]

Supplementary Materials

Table S1. All statistical analyses with significant differences in Figs.

position	Bonferroni's multiple comparisons test	N1	N2	Mean Diff	95%CI	Adjusted P value
Fig.2b	S1-D14 vs S2-D14	17	15	-1.482	-1.863, -1.101	<0.0001
	S1-D14 vs S2-D28	17	52	-1.525	-1.863, -1.225	<0.0001
	S1-D28 vs S2-D14	29	15	-1.291	-1.632, -0.949	<0.0001
	S1-D28 vs S2-D28	29	52	-1.334	-1.583, -1.085	<0.0001
	S2-D0 vs S2-D14	66	15	-1.510	-1.817, -1.202	<0.0001
	S2-D0 vs S2-D28	66	52	-1.553	-1.752, -1.354	<0.0001
Fig.2e	S1-D14 vs S1-D28	12	29	-0.603	-0.968, -0.238	<0.0001
	S1-D14 vs S2-D0	12	68	-0.348	-0.681, -0.014	0.0343
	S1-D14 vs S2-D14	12	15	-2.012	-2.424, -1.599	<0.0001
	S1-D14 vs S2-D28	12	52	-2.213	-2.553, -1.872	<0.0001
	S1-D28 vs S2-D0	29	68	0.256	0.020, 0.492	0.0242
	S1-D28 vs S2-D14	29	15	-1.408	-1.747, -1.070	<0.0001
	S1-D28 vs S2-D28	29	52	-1.609	-1.856, -1.363	<0.0001
	S2-D0 vs S2-D14	68	15	-1.664	-1.968, -1.360	<0.0001
	S2-D0 vs S2-D28	68	52	-1.865	-2.061, -1.669	<0.0001
Fig.3c	A vs D	113	23	0.461	0.053 to 0.869	0.0178
Fig.3d	A vs D	111	23	0.583	0.190 to 0.976	0.0007
Fig.4c	Cecolin 2 vs Gardasil 4	14	16	0.778	0.145 to 1.411	0.0058
	Cecolin 2 vs Gardasil 9	14	162	0.605	0.123 to 1.086	0.0045
	Cecolin 2+ vs Gardasil 4	66	16	0.619	0.137 to 1.100	0.0034
	Cecolin 2+ vs Gardasil 9	66	162	0.445	0.193 to 0.700	<0.0001
	Cervarix 2 vs	21	16	0.750	0.176 to	0.0026

	Gardasil 4				1.324	
	Cervarix 2 vs Gardasil 9	21	162	0.576	0.175 to 0.977	0.0006
Fig.4d	Cecolin 2 vs Gardasil 4	13	15	1.194	0.606 to 1.782	<0.0001
	Cecolin 2 vs Gardasil 9	13	159	0.880	0.432 to 1.328	<0.0001
	Cecolin 2+ vs Gardasil 4	65	15	1.428	0.983 to 1.873	<0.0001
	Cecolin 2+ vs Gardasil 9	65	159	1.114	0.886 to 1.343	<0.0001
	Cervarix 2 vs Gardasil 4	21	15	1.083	0.558 to 1.608	<0.0001
	Cervarix 2 vs Gardasil 9	21	159	0.769	0.408 to 1.129	<0.0001

Table S2. The GMTs and HPV NAb seroprevalence of the different stages of the cohort.

	HPV type	S1			S2		
		D0	D14	D28	D0	D14	D28
GMT [95%CI]	HPV 16	-	182 [134, 249]	283 [194, 413]	171 [141, 208]	5526 [2845, 10732]	6107 [4813, 7749]
	HPV 18	-	83 [48, 142]	333 [228, 485]	185 [152, 225]	8523 [4651, 15616]	13535 [10927, 16766]
Seroprevalence (%, Positive/Total)	HPV 16	0 (0/38)	94.4 (17/18)	93.5 (29/31)	94.3 (66/70)	100 (15/15)	100 (52/52)
	HPV 18	0 (0/38)	66.7 (12/18)	93.5 (29/31)	97.1 (68/70)	100 (15/15)	100 (52/52)
	HPV 6	0 (0/38)	0 (0/18)	0 (0/31)	0 (0/70)	0 (0/15)	0 (0/52)
	HPV 11	0 (0/38)	0 (0/18)	0 (0/31)	0 (0/70)	0 (0/15)	0 (0/52)
	HPV 31	0 (0/38)	11.1 (2/18)	22.6 (7/31)	0 (0/70)	13.3 (2/15)	15.4 (8/52)
	HPV 33	0 (0/38)	11.1 (2/18)	6.5 (2/31)	0 (0/70)	33.3 (5/15)	32.7 (17/52)
	HPV 45	0 (0/38)	0 (0/18)	0 (0/31)	0 (0/70)	20 (3/15)	5.8 (3/52)
	HPV 52	0 (0/38)	0 (0/18)	0 (0/31)	0 (0/70)	0 (0/15)	5.8 (3/52)
	HPV 58	0 (0/38)	0 (0/18)	25.8 (8/31)	0 (0/70)	6.7 (1/15)	7.7 (4/52)

All participants in S1-D0 tested negative (titer < 40), hence no data were shown.

Table S3. Adverse events reported during Days 0-28 post-dose 1, 2 across all vaccination visits.

symptoms		N Total=87	Incidence % [95CI]
injection site reactions	skin swelling	6	6.90 [3.20, 14.24]
	Skin firmness	2	2.30 [0.41, 8.00]
	skin itch	6	6.90 [3.20, 14.24]
	Subsequent pain	6	6.90 [3.20, 14.24]
adverse events	Nausea	1	1.15 [0.06, 6.23]
	Muscle ache	3	3.45 [0.94, 9.65]
	Headache	2	2.30 [0.41, 8.00]
	Tiredness	3	3.45 [0.94, 9.65]
	fever	2	3.45 [0.94, 9.65]

All adverse reactions were reported by the parents of the subjects during next visit.

Table S4. The GMTs of HPV16&HPV18 NAbs in the several groups.

vaccine	N	PBNA-GMT [95%CI]	
		HPV16	HPV18
Cecolin 2	14	9739 [3677, 25796]	6903 [3437, 13863]
Cecolin 2+	101	6743 [5163, 8807]	11830 [9486, 14754]
Cervarix 2	21	9122 [5451, 15264]	5342 [3309, 8625]
Gardasil 4	17	1623 [789, 3338]	442 [176, 1109]
Gardasil 9	163	2420 [1909, 3607]	910 [733, 1129]
Vaccinated*	215		
A	113	3886 [2878, 5247]	1670 [1255, 2223]
B	44	2748 [1814, 4163]	1018 [663, 1562]
C	34	2104 [1213, 3652]	830 [488, 1410]
D	24	1344 [703, 2571]	436 [226, 844]
unvaccinated	50	-	-

Cecolin 2+ was composed of samples from the adults and samples of the S2-D28. All adult vaccine recipients were divided into four groups based on the time after the last dose (A:0.1-12, B:12-24, C:24-36 D:36-75 months). All participants in the control group tested negative (titer < 40), hence no data were shown. *Not including the females aged 9-14 years.

Table S5. Seroprevalence of nine HPV types NAbs in five vaccine groups.

	Seroprevalence (Positive/Total)				
	Cecolin 2	Cecolin 2+	Cervarix 2	Gardasil 4	Gardasil 9
HPV16	100% (14/14)	100% (66/66)	100% (21/21)	94.12% (16/17)	99.39% (162/163)
HPV18	92.86% (13/14)	98.48% (65/66)	100% (21/21)	88.24% (15/17)	97.55% (159/163)
HPV6	7.14% (1/14)	1.52% (1/66)	9.52% (2/21)	100% (17/17)	98.77% (161/163)
HPV11	7.14% (1/14)	1.52% (1/66)	4.76% (1/21)	94.12% (16/17)	98.77% (161/163)
HPV31	71.43% (10/14)	27.27% (18/66)	28.57% (6/21)	5.88% (1/17)	96.32% (157/163)
HPV33	7.14% (1/14)	27.27% (18/66)	9.52% (2/21)	0% (0/17)	95.09% (155/163)
HPV45	14.29% (2/14)	7.58% (5/66)	4.76% (1/21)	0% (0/17)	84.05% (137/163)
HPV52	0% (0/14)	4.55% (3/66)	9.52% (2/21)	0% (0/17)	85.90% (140/163)
HPV58	0% (0/14)	6.06% (4/66)	9.52% (2/21)	0% (0/17)	94.48% (154/163)

Table S6. Plasmids and Cell used in this study.

Plasmids/Cell		Function	RRID
reporter plasmids	pCMV-C-EGFP	Enhanced Green Fluorescent Protein	-
	pRwB	Red Fluorescent Protein	Addgene_48734
HPV L1L2 expressing plasmids	p16sheLL	HPV16 L1L2 VLP	Addgene_37320
	p18sheLL	HPV18 L1L2 VLP	Addgene_37321
	p6sheLLr	HPV6 L1L2 VLP	Addgene_37318
	pVITRO-HPV11L1L2	HPV11 L1L2 VLP	Addgene_52590
	p31sheLL	HPV31 L1L2 VLP	Addgene_37322
	pVITRO-HPV33 L1L2	HPV33 L1L2 VLP	Addgene_52493
	p45sheLL	HPV45 L1L2 VLP	Addgene_37323
	p52sheLL	HPV52 L1L2 VLP	Addgene_46950
	p58sheLL	HPV58 L1L2 VLP	Addgene_37324
Cell	HEK293FT	Transfection Vector	CVCL_6911
